# Health-related quality of life and associated factors among older refugees from Nagorno-Karabakh residing in Kotayk province of Armenia

**DOI:** 10.3389/fpubh.2025.1718203

**Published:** 2026-01-12

**Authors:** Diana Muradyan, Aida Giloyan, Tsovinar Harutyunyan, Varduhi Petrosyan

**Affiliations:** 1 Turpanjian College of Health Sciences, American University of Armenia, Yerevan, Armenia; 2 Garo Meghrigian Institute for Preventive Ophthalmology, Turpanjian College of Health Sciences, American University of Armenia, Yerevan, Armenia

**Keywords:** health related quality of life, mental health component, non-communicable diseases, physical health component, refugees

## Abstract

**Introduction:**

This study aimed to explore factors associated with health-related quality of life (HRQoL) among refugees aged 50 years and older from Nagorno-Karabakh residing in Armenia.

**Methods:**

We used a structured interviewer-administered questionnaire to collect data on socio-demographic characteristics, presence of non-communicable diseases (NCDs), visual impairment (VI), eye diseases, smoking, sleep disorders, HRQoL, and healthcare utilization in a sample of 197 participants. The Short-Form Health Survey (SF-36) questionnaire was used to measure the physical health component (PHC) and mental health component (MHC) of HRQoL. A comprehensive ophthalmic examination was carried out.

**Results:**

The mean age of participants was 64.84 (SD = 9.28), with 65.48% being female. The mean MHC and PHC scores were 59.37 (SD = 20.04), and 59.4 (SD = 20.1) to 63.57 (SD = 26.28), respectively. Hypertension, bone/joint diseases, and heart diseases were the most frequently reported diseases in our sample. In the adjusted linear regression analysis, age, sleep disorders, respiratory diseases, bone/joint diseases, and diabetes were significantly associated with both PHC and MHC scores of SF-36. Heart disease was associated with PHC only. Having more than one NCD was negatively associated with PHC and MHC. In the domain specific adjusted analysis, bone/joint diseases and diabetes, were significantly associated with physical functioning, general health, and energy/fatigue domains of the PHC and MHC components of HRQoL.

**Conclusion:**

Governmental support and interventions targeting older refugees and those with more than one NCD and sleep disorders may be beneficial. Follow-up assessments should monitor variations in both PHC and MHC scores among refugees, as they may vary over time in response to the challenges associated with displacement.

## Introduction

According to the United Nations Refugee Agency estimates, in 2023, the number of forcibly displaced people globally comprised 117.3 million, with 31.6 million of them categorized as refugees (1). Following a military offensive by Azerbaijan on September 19–20, 2023 the entire population of Nagorno-Karabakh (NK), a de-facto state inhabited by ethnic Armenians, fled to Armenia (2, 3). More than 100,000 refugees were settled in different regions of Armenia, including the capital city Yerevan (38%), and the provinces of Syunik (16%), Kotayk (8%), and Ararat (7%) where they had access to services, such as emergency shelter and housing, food and basic supplies, medical assistance, mental health and psychological services, employment and multipurpose cash assistance (4, 5). Before the forced displacement, the population of NK was in a 9-month blockade imposed by Azerbaijan, which deprived NK Armenians of access to food, medication, and other goods (3). These all led to malnutrition and a decline in health due to severe shortages of food, medical supplies, and vaccines (2, 6).

Forced migration is associated with a number of threats to physical and mental health and well-being (7). Refugees can face dangerous travel conditions, overcrowded transportation, threats, like shooting or bombing, which can lead to injuries or disabilities (8). Forcibly displaced people experience a lack of access to healthcare, medications, maternal care, and prevention services, such as vaccination (9). Specifically, refugees with non-communicable diseases (NCDs) may lose access to necessary medications and treatments, resulting in poorly controlled conditions and long-term complications (10). Inadequate healthcare infrastructure in host countries can lead to low-quality of care (11). Also, forced displacement can result in the shortage of food, leading to malnutrition (12).

Recent studies report the development of symptoms of depression and anxiety even at 3 years after forced displacement (13–15). Forced migration may include separation from family members, exposure to violence, and the loss of loved ones, which may lead to mental disorders, specifically to anxiety, posttraumatic stress disorders (PTSD), and depression among unaccompanied refugee minors (16, 17). Based on stress theories, Miller and Rasmussen (18) suggested that persistent daily stressors may deplete coping mechanisms and impede recovery after trauma (19). In contrast, several studies showed the resilience of children and adults in the areas of armed conflict, which was explained by having good mental health as a result of adaptive mechanisms (20, 21).

Several studies have been published on the refugees’ health-related quality of life (HRQoL) in high-income countries. Most of them have shown generally lower quality of life among refugees compared to that of the host country population (22–24). Evidence from Finland, Sweden, and other high-income countries shows that older refugees often experience poorer physical and mental health than comparably aged residents, including higher levels of anxiety, depression, and functional limitations (25, 26).

A range of factors has been identified as influencing HRQoL in refugee populations, including prior trauma exposure, mental health symptoms, NCDs, and limited social support (27–30). Also, it was shown that refugee women reported more physical and mental issues than men (15, 31). Older refugees reported more mental health issues (32) and poor physical functioning than younger ones (27). Other factors associated with HRQoL among refugees are marital status (33, 34), education (33, 34), occupation (33), employment status (35), and income (35).

In addition, sleep disturbances which are common among refugees due to psychological distress, comorbid conditions, or post-traumatic stress have been consistently associated with poorer HRQoL outcomes (23, 36–38).

It has been shown that lower HRQoL scores might be associated with more frequent healthcare utilization (29). Issues, mostly thought to be caused by limited access to healthcare following displacement, have also been noted. For example, eye health might deteriorate after displacement (39).

Individuals aged 50 years and above often have a higher burden of NCDs, poorer physical functioning, and greater dependency on continuous medical care. Studies show that older refugees experience more mental health problems and reduced physical functioning than younger refugees (27). Despite this, older adults remain underrepresented in refugee health research, including HRQoL studies.

Although the recent displacement from NK has generated substantial humanitarian concerns, there is little evidence on the health status and HRQoL of this population in the literature. In particular, the needs of older adults who often carry a higher burden of chronic disease, functional limitations, and psychosocial stressors remain largely undocumented. To address this gap, we aimed to explore potential risk factors of HRQoL among NK refugees aged 50 years and older currently residing in Kotayk province of Armenia.

## Methods and materials

### Study population and data collection

The current study was conducted in the scope of a larger project, which included free screening and distribution of eyeglasses and medications to forcibly displayed population by the Meghrigian Institute for Preventive Ophthalmology at the American University of Armenia (40). We recruited forcibly displaced individuals aged 50 and above from NK, who were residing in Kotayk province of Armenia, which is one of the provinces in geographic proximity to the capital Yerevan. To access the target population, the research team visited previously selected locations in Kotayk with the highest concentration of forcibly displaced people. An assigned person at each location coordinated and scheduled advance appointments for participation in the eye screening and the survey. All willing participants underwent ophthalmic screenings. The exclusion criteria for the survey included hearing and cognitive impairments that could hinder the administration of the questionnaire. The final non-random sample included 197 people aged 50 years and above, who participated in both the survey and ophthalmic examinations. Oral consent was obtained from the patients before starting the interviews.

The survey team consisted of an ophthalmologist, an ophthalmic nurse, and an interviewer. Before starting the survey, the team underwent training on survey administration. The data was collected in October–November 2023.

### Study instrument

A structured interviewer-administered questionnaire contained questions regarding the respondents’ age, gender, education, social and marital status, monthly expenditure, occupation, presence of NCDs, visual impairment (VI) and eye diseases, smoking, sleep disorders, HRQoL, and healthcare utilization.

To assess self-reported NCDs participants were asked to indicate any chronic health problems that they presently have with response options including hypertension, bone/ joint, heart, kidney, gastro-intestinal diseases, and respiratory diseases/ asthma, as well as the “other” option. Participants were allowed to list more than one condition. To assess sleep disorders, participants were asked if they had experienced sleep problems in the past 30 days. The response options were: “never”, “seldom,” “sometimes,” “often,” and “very often.” Short-Form Health Survey (SF-36) tool, which is validated and translated into different languages (41), including Armenian, was used to assess participants’ HRQoL (42). The tool consists of 36 items subdivided into eight domains. Responses are converted to 0–100 scores and averaged, with higher values indicating better HRQoL (43). The domains including “physical functioning,” “role limitations due to physical health,” “bodily pain” and “general health” constituted the Physical Health Component (PHC), while “social functioning,” “role limitations due to emotional problems,” “emotional well-being,” and “energy/ fatigue” represented the Mental Health Component (MHC) (44).

All interviewers participated in a training program prior to the start of data collection. Interviewers were introduced to the purpose of the study, target population, and study protocol. Each questionnaire item was reviewed in depth and discussed with interviewers. Also, they were trained on ethical principles, including informed consent, confidentiality, voluntary participation, and respectful communication with older and potentially vulnerable participants. The interviewers used tablets with the Alchemer survey tool to administer the questionnaire. Responses were entered electronically in real time, ensuring accurate data capture and reducing transcription errors. Interviewers participated in a small pilot test to practice real field conditions. Feedback from the pilot was used to further refine interviewer performance and clarify procedures. During data collection, field supervisors monitored interviews, reviewed data daily, and provided continuous feedback to maintain quality and consistency. The interviews took 15 min, on average.

### Ophthalmic examination

After the interview, the research team performed a comprehensive ophthalmic examination.

Visual acuity was measured using a Golovin-Sivtsev (Snellen equivalent) chart. Intraocular pressure was measured and dilated eye fundus examination was performed through an ophthalmoscope. The study applied World Health Organization International Classification of Diseases (ICD - 11) criteria to define eye diseases including cataract, glaucoma suspect, diabetic retinopathy, and age-related macular degeneration (45).

Based on the severity level of visual acuity, VI was classified into four groups: mild VI was defined as a presenting visual acuity (PVA) of less than 6/12 but greater than or equal to 6/18; moderate VI included a PVA of less than 6/18 but greater than or equal to 6/60; severe VI included PVA of less than 6/60 but greater than or equal to 3/60. Blindness was categorized as a PVA lower than 3/60 (46).

### Study variables

PHC and MHC scores of SF-36 were the outcome variables. Age, gender, education (incomplete secondary and secondary, college and higher), self-assessed social status (substantially below average/below average, average/little above/substantially above average), marital status (married/divorced, widow/single), occupation (employed, unemployed, retired), smoking status (never smokers, current smokers, ever smokers), presence of NCDs (hypertension, heart, respiratory/asthma, GIT, cancer, kidney and bone/joint diseases), and the number of NCDs (no disease, one disease, and two and more NCDs), utilization of medical services since moving to Armenia (yes/no), eye diseases glaucoma, cataract, diabetic retinopathy, age –related macular degeneration (AMD), and VI (yes/no) were the independent variables.

### Analysis

The data was analyzed using SPSS Statistical Package version 21.0 (SPSS inc., Chicago, IL, USA) and STATA/SE 13 version (StataCorp. 2013. Stata Statistical Software: Release 13. College Station, TX: StataCorp LP.). A descriptive analysis was conducted, using means and standard deviations for continuous variables and percentages for categorical variables. Univariate linear regression analysis explored associations between each independent variable and PHC and MHC scores. Those independent variables that showed a significant association with the outcome variables at a *p*-value less than 0.05 in a univariate analysis were included in the multivariable models (47). A similar strategy was used for exploring the impact of all independent variables on specific SF-36 domains. To assess the link between risk factors and PHC and MHC scores of SF-36, we developed two models: the first model incorporated all independent variables of interest, including individual NCDs, VI, and eye diseases. The second model included the number of NCDs variable, excluding individual NCDs due to their significant correlation with one another. Collinearity was checked and no single variable in the models exceeded the VIF factor over 5.0. Assumptions underlying linear regression were examined by assessing the normality of residuals with Q–Q plots and evaluating homoscedasticity through residual-versus-fitted plots.

### Ethical consideration

The Institutional Review Board of the American University of Armenia approved the research protocol (PROTOCOL #: AUA-2023-018). The oral consent form was provided to all participants before the beginning of the study. Oral consent was used to avoid discomfort or safety concerns related to signing documents in this vulnerable refugee population.

## Results

### Descriptive analysis

The mean age of participants was 64.84 (SD = 9.28), ranging from 50 to 95. Around 70% of participants were women. More than half of the participants had incomplete or complete secondary education. Around 50% of study participants assessed their standard of living as average, while 21.32% had a somewhat below average and 13.20% had a substantially below-average family standard of living. Hypertension (55.33%), bone/ joint diseases (34.01%), and heart diseases (24.87%) were the most frequently reported diseases in our sample. Normal vision was observed in 74.62% of the participants, whereas 24.87% experienced moderate to severe VI, and 0.51% were blind. The mean PHC score was 63.57 (SD = 26.28) and the mean MHC score was 59.37 (SD = 20.04). The median PHC score was 76.25, with two modes observed at 83.75 and 87.50. The median MHC score was 68.63, with a mode of 69.50. Only 27.92% of the study participants have used healthcare services after displacement (Table 1).

**Table 1:** Demographic characteristics of the study participants.

Variables	Total, *n* (%)
Age (Mean, SD)	64.84 (9.28)
Gender	
Male	68 (34.52)
Female	129 (65.48)
Education	
Incomplete secondary and secondary (> 10 years)	116 (58.88)
College and higher (< 10 years)	81 (41.12)
Family standard of living	
Substantially below average	26 (13.20)
Below average	42 (21.32)
Average	99 (50.25)
Little above average	1 (0.51)
Substantially above average	1 (0.51)
Marital status	
Married	128 (65.97)
Divorced	8 (4.06)
Widow	57 (28.93)
Single	4 (2.03)
Family expenditure [Armenian dram (AMD)]	
Less than 100,000	102 (86.44)
101,000-200,000	16 (13.56)
Occupation	
Employed	5 (2.54)
Unemployed	75 (38.07)
Retired	117 (59.39)
Smoking status	
Never smokers	135 (68.53)
Current smokers	42 (21.32)
Ever smokers	20 (10.15)
Sleep disorder	
Never	16 (8.12)
Seldom/sometimes	85 (43.15)
Often/very often	96 (48.73)
Visual impairment (VI)	
Normal	147 (74.62)
Mild/Moderate/Severe (MMS)	49 (24.87)
Blindness	1 (0.51)
Non-communicable diseases (NCDs)	
Hypertension	109 (55.33)
Bone/joint disease	67 (34.01)
Heart disease	49 (24.87)
Diabetes	30 (15.23)
Gastrointestinal disease	10 (5.08)
Respiratory disease/ asthma	8 (4.06)
Renal disease	14 (7.11)
Other NCDs*	26 (13.20)
SF-36 (Mean, SD)	
Physical functioning	64.82 (32.89)
Role limitations due to physical health	64.72 (47.77)
Role limitations due to emotional problems	64.97 (47.83)
Energy/fatigue	47.49 (14.08)
Emotional well-being	37.25 (16.36)
Social functioning	87.75 (20.36)
Bodily pain	83.27 (25.76)
General health	41.46 (13.48)
Physical health component (PHC) score (Mean, SD)	63.57 (26.28)
Mental health component (MHC) score (Mean, SD)	59.37 (20.04)
Number of NCDs	
No NCD	47 (23.86)
One NCD	52 (26.40)
More than one NCDs	98 (49.75)
Use of medical service	55 (27.92)
Type of medical service	
Primary healthcare	22 (39.29)
Narrow specialist	29 (51.79)
Emergency services	5 (8.93)

*Other NCDs include cancer, neurological, vascular, endocrine, skin, infections, hernia, allergic diseases and benign tumor.

### Simple and multivariable linear regression analysis

In the unadjusted linear regression analysis age, marital status, sleep disorders, use of medical service, VI, hypertension, heart, respiratory, bones and joint diseases, diabetes, and the number of NCDs were negatively associated with both PHC and MHC scores of HRQoL. Gender, smoking status, and eye diseases were negatively associated with PHC score only (Tables 2, 3).

**Table 2:** Unadjusted and adjusted linear regression analysis of factors associated with physical health component scores of SF-36 among refugees aged 50 years and older from Nagorno-Karabakh residing in Kotayk province of Armenia.

	Physical health component			
	Unadjusted		Adjusted	
Variables	B (95% CI)	*P* value	B (95% CI)	*P* value
Age	−1.08 (−1.47; −0.70)	**<0.001**	−0.53 (−0.98; −0.08)	**0.023**
Gender				
Male	1.0		1.0	
Female	−7.80 (−15.51; −0.09)	**0.047**	4.67 (−7.87; 17.21)	0.463
Education				
Incomplete secondary and secondary (> 10 years)	1.0			
College and higher (< 10 years)	7.21 (−0.24; 14.67)	0.058		
Social status				
Substantially below average/ below average	1.0			
Average/little above/substantially above average	7.10 (−0.65; 14.84)	0.072		
Marital status				
Married	1.0		1.0	
Divorced	−11.14 (−29.83; 7.55)	0.241	−5.87 (−21.70; 9.95)	0.465
Widow	−10.52 (−18.69; −2.36)	**0.012**	−4.56 (−12.49; 3.37)	0.258
Single	−0.67 (−26.72; 25.37)	0.959	9.49 (−11.20; 30.17)	0.366
Occupation				
Employed	1.0			
Unemployed	1.68 (−20.10; 23.45)	0.880		
Retired	−21.16 (−42.69; 0.37)	0.054		
Smoking status				
Never smokers	1.0		1.0	
Current smokers	6.45 (−2.62; 15.52)	0.163	3.51 (−9.74; 16.75)	0.602
Ever smokers	13.45 (1.15; 25.76)	**0.032**	12.67 (−1.75; 27.09)	0.085
Sleep disorder				
Never	1.0		1.0	
Seldom/sometimes	−7.79 (−20.40; 4.81)	0.224	−2.54 (−14.31; 9.24)	0.671
Often/very often	−30.35 (−42.84; −17.85)	**<0.001**	−14.93 (−26.96; −2.90)	**0.015**
Use of medical service				
No	1.0		1.0	
Yes	−14.52 (−22.52; −6.52)	**<0.001**	−3.64 (−11.12; 3.83)	0.338
Non-communicable diseases (NCDs)				
Hypertension	−19.45 (−26.38;-12.53)	**<0.001**	−2.49 (−9.64; 4.67)	0.493
Heart disease	−26.62 (−34.32; 18.92)	**<0.001**	−12.26 (−20.60; −3.92)	**0.004**
Respiratory diseases/asthma	−23.83 (−42.29;-5.38)	**0.042**	−25.27 (−46.02; −4.51)	**0.017**
Gastrointestinal disease	9.61 (−7.21; 26.42)	0.261		
Kidney disease	−6.53 (−20.92; 7.85)	0.371		
Bone/joint disease	−22.27 (−29.43; −15.12)	**<0.001**	−11.62 (−18.76; −4.47)	**0.002**
Diabetes	−14.31 (−24.42; −4.21)	**0.006**	−12.29 (−21.66; −2.92)	**0.010**
Visual impairment (VI)				
No	1.0		1.0	
Yes	−11.65 (−20.00; −3.30)	**0.006**	−4.01 (−11.42; 3.40)	0.286
Eye diseases*				
No	1.0		1.0	
Yes	−12.08 (−19.59; 4.56)	**0.002**	−0.93 (−8.79; 6.94)	0.816
Number of NCDs^1^				
No NCDs	1.0		1.0	
One NCD	−13.84 (−22.93; −4.75)	**0.003**	−9.87 (−18.71; −1.04)	**0.029**
More than one NCD	−31.52 (−39.53; −23.50)	**<0.001**	−18.41 (−27.13; −9.69)	**<0.001**

^1^The number of NCDs were included in the main model while individual NCDs were removed.

*Eye diseases include glaucoma, cataract, diabetic retinopathy, age—related macular degeneration.

Bold values indicate statistically significant associations (*p* < 0.05).

**Table 3:** Unadjusted and adjusted linear regression analysis of factors associated with mental health component scores of SF-36 among refugees aged 50 years and older from Nagorno-Karabakh residing in Kotayk province of Armenia.

	Mental health component			
	Unadjusted		Adjusted	
Variables	B (95% CI)	*P* value	B (95% CI)	*P* value
Age	−0.76 (−1.06; −0.47)	**<0.001**	−0.38 (−0.67; −0.09)	**0.010**
Gender				
Male	1.0			
Female	−3.75 (−9.66; 2.17)	0.213		
Education				
Incomplete secondary and secondary (> 10 years)	1.0			
College and higher (< 10 years)	3.85 (−1.86; 9.56)	0.185		
Social status				
Substantially below average/below average	1.0			
Average/little above/substantially above average	4.52 (−1.43; 10.47)	0.135		
Marital status				
Married	1.0		1.0	
Divorced	−0.82 (−15.08; 13.45)	0.910	1.74 (−10.71; 14.20)	0.783
Widow	−8.07 (−14.30; −1.84)	**0.011**	−3.17 (−8.98; 2.65)	0.284
Single	1.45 (−18.43; 21.33)	0.886	7.70 (−8.58; 23.98)	0.352
Occupation				
Employed	1.0			
Unemployed	1.31 (−15.40; 18.03)	0.877		
Retired	−15.54 (−32.07; 0.98)	0.065		
Smoking status				
Never smokers	1.0			
Current smokers	2.88 (−4.10; 9.85)	0.417		
Ever smokers	6.88 (−2.58; 16.34)	0.153		
Sleep disorder				
Never	1.0		1.0	
Seldom/sometimes	−4.56 (−14.06; 4.94)	0.345	−1.72 (−10.94; 7.51)	0.714
Often/very often	−22.87 (−32.28; −13.46)	**<0.001**	−13.14 (−22.56; −3.71)	**0.007**
Use of medical service				
No	1.0		1.0	
Yes	−10.59 (−16.70; −4.48)	**0.001**	−3.92 (−9.75; 1.92)	0.187
Non-communicable diseases (NCDs)				
Hypertension	−13.94 (−19.27; −8.62)	**<0.001**	−2.73 (−8.22; 2.76)	0.328
Heart disease	−17.56 (−23.60; −11.52)	**<0.001**	−6.18 (−12.73; 0.38)	0.064
Respiratory diseases/asthma	−20.02 (−34.04; −6.01)	**0.005**	−16.58 (−32.83; −0.33)	**0.046**
Gastrointestinal disease	7.18 (−5.63; 20.00)	0.270		
Kidney disease	−7.19 (−18.13; 3.75)	0.196		
Bone/joint disease	−15.28 (−20.83; −9.73)	**<0.001**	−7.30 (−12.78; −1.81)	**0.009**
Diabetes	−9.39 (−17.14; −1.65)	**0.018**	−7.91 (−15.30; −0.51)	**0.036**
Visual impairment (VI)				
No	1.0		1.0	
Yes	−9.16 (−15.52; −2.80)	**0.005**	−3.34 (−9.12; 2.44)	0.255
Eye diseases*				
No	1.0			
Yes	−6.44 (−13.61; 0.73)	0.078		
Number of NCDs^1^				
No NCDs	1.0		1.0	
One NCD	−10.43 (−17.49; −3.37)	**0.004**	−6.64 (−13.42; 0.13)	0.054
More than one NCD	−22.68 (−28.91; −16.45)	**<0.001**	−12.51 (−19.12; −5.90)	**<0.001**

^1^The number of NCDs were included in the main model while individual NCDs were removed.

*Eye diseases include glaucoma, cataract, diabetic retinopathy, age—related macular degeneration.

Bold values indicate statistically significant associations (*p* < 0.05).

In the adjusted linear regression analysis in the first model age (PHC; *p* = 0.023 MHC; *p* = 0.010), sleep disorders (PHC; *p* = 0.015 MHC; *p* = 0.007), respiratory diseases/ asthma (PHC; *p* = 0.017; MHC; *p* = 0.046), bone/ joint diseases (PHC; *p* = 0.002; MHC; *p* = 0.009) and diabetes (PHC; p = 0.010; MHC; *p* = 0.036) were significantly associated with both PHC and MHC scores of SF-36. Heart diseases (*p* = 0.004) was associated with PHC only. In the second model, more than one NCD (PHC; *p* < 0.001; MHC; *p* < 0.001) was associated with both PHC and MHC (Tables 2, 3).

In the adjusted linear regression analysis assessing factors associated with each domain of HRQoL showed that sleep disorder was associated with physical functioning, role limitation due to physical and emotional health, and emotional well-being. Heart disease was negatively associated with physical functioning, role limitations due to physical health, general health, and role limitations due to emotional problems. Bone/joint diseases were negatively associated with all domains, except role limitations due to physical health, role limitations due to emotional problems, and emotional well-being. Diabetes was negatively associated with all domains, except pain, emotional well- being and social functioning. (Table 4).

**Table 4:** The results of adjusted linear regression of risk factors associated with each domain of HRQoL among refugees aged 50 years and older from Nagorno-Karabakh residing in Kotayk province of Armenia.

	Physical health component^a^				Mental health component^b^			
Variables	Physical functioning	Role limitation physical	Pain	General health	Role limitation emotional	Energy/fatigue	Emotional well- being	Social functioning
	B	B	B	B	B	B	B	B
Sleep disorder								
Never	1.0	1.0	1.0	1.0	1.0	1.0	1.0	1.0
Seldom/sometimes	−3.69	−5.79	4.57	**−**0.87	−7.95	−0.68	−2.44	6.53
Often/very often	−17.29*	−27.09*	−5.88	**−**5.59	−30.45*	**−**5.46	−12.00*	−1.67
Non-communicable diseases (NCDs)								
Hypertension	−1.65	−3.76	−0.57	−4.23*	−2.79	−2.58	−2.80	−3.32
Heart disease	−18.10**	−30.34***	2.64	−4.60*	−29.34***	−0.004	1.87	1.70
Respiratory diseases/asthma	−21.74	−39.32	−28.22*	−9.19	−35.87	−12.05*	4.73	−20.11*
Bone/joint disease	−13.36**	−9.39	−16.07***	−7.66***	−10.66	−6.50**	−2.63	−9.02**
Diabetes	−20.60**	−19.04*	−5.44	−7.87**	−20.00*	−8.13**	−4.32	−2.29
Visual impairment (VI)								
No	1.0	1.0	1.0	1.0	1.0	1.0	1.0	1.0
Yes	−1.85	−10.76	−1.61	−0.17	−12.36	−1.65	1.87	0.19
Eye diseases								
No	1.0	1.0	1.0	1.0				
Yes	0.67	0.87	−5.86	−1.86				

*p < 0.05; **p < 0.01; ***P < 0.001.

^a^Adjusted for age, gender, marital status, smoking status, use of medical services, and other NCDs.

^b^Adjusted for age, marital status, and use of medical services.

Bold values indicate statistically significant associations (*p* < 0.05).

## Discussion

This study assessed PHC and MHC of HRQoL and factors associated with these components among refugees from NK residing in Kotayk province of Armenia.

As expected, lower HRQoL of refugees was significantly associated with older age, presence of multiple NCDs, sleep disorders, and chronic conditions such as heart disease, bone and joint diseases, respiratory diseases, and diabetes in our study. In our sample, PHC and MHC scores were 63.57 and 59.37, respectively. These scores are unexpectedly higher than those recorded in similar studies, including surveys conducted in Greece (48) and Germany (49) among refugees, where the PHC and MHC scores were 43.9 and 53.4 in Greece, and 39.5 and 47.9 in Germany, respectively. We hypothesized that, despite the hardships of displacement, the NK refugees may experience a higher quality of life compared to other similar populations due to their easier cultural integration into host communities because they are Armenians and have the same language and traditions as the host population (50). This can decrease the stress of adjusting to a new environment and help to reduce feelings of isolation and stress (51). However, this assumption was not assessed and information on language barriers, discrimination, or social integration was not collected in our study. The difference in PHC and MHC composite scores between our and previous studies may also be explained by methodological variations, as our study employed RAND-36 composite scores (52), while other studies may have used alternative scoring techniques or reference population.

This study was conducted during the same year that the refugees from NK were displaced to Armenia, thereby HRQoL was measured immediately after the forced migration. Unlike studies conducted several years after the displacement (53, 54), our findings presented an acute phase and may not represent the long-term evolution of HRQoL. Previous studies suggest that prolonged exposure to unstable living conditions, psychosocial stressors, and unmet health and social needs may negatively affect HRQoL over time (55).

In our sample, 43.15% of participants reported experiencing sleep disorders seldom or sometimes, while 48.73% reported sleep disorders often or very often. This is substantially higher compared to the rates obtained from nursing home residents in Armenia (28.8 and 39.6%, correspondingly) (56). In our study, participants experiencing sleep disorders often/ very often reported significantly lower scores in both PHC and MHC of HRQoL than those without sleep disorders. There is limited data available on sleep disorders and HRQoL among refugees; however, several studies assessed this link in the general population. Stranges et al. (57) and Darchia et al. (58) reported a significant association between sleep duration, sleep quality, and PHC and MHC domain of HRQoL among 45–69 years old adults in a cross-cultural comparison study between the United Kingdom and the US, and among 20–60 years old residents in Tbilisi and Kutaisi cities in Georgia. However, another study conducted in the US among middle-aged residents did not find an association between sleep duration and HRQoL (59). Poor sleep increases fatigue, diminishes concentration, and increases the risk of depression (60, 61). It also influences mood, productivity, and social interactions, leading to decreased overall life satisfaction (62). These factors may diminish emotional and physical well-being and adversely affect HRQoL (63).

Only 27.92% of refugees have utilized medical services after displacement. Financial constraints, including the cost of medications, diagnostic tests, and treatment, are a well-documented barrier to healthcare utilization among refugees (64). Early post-displacement priorities, such as securing food and shelter, may also reduce healthcare use, particularly for non-urgent conditions (65). Living in remote areas may create additional challenges in accessing healthcare services (64). An overwhelmed healthcare system might have also influenced refugees’ healthcare utilization patterns.

In our study age was negatively associated with both PHC and MHC scores. Several authors reported a significant association between age and poor HRQoL, which aligns with our study results (28, 48). Another cross-sectional study conducted among asylum seekers in Germany found that age was associated with both MHC and PHC scores of HRQoL (48). This can be explained by the fact that older participants might find adjusting to new environmental conditions more challenging (28). Similar associations have been reported in the general population, where aging is commonly linked to reduced mobility, limitations in daily activities, chronic conditions, social isolation, and changes in physical health, and loss of family members which can benegatively associated with both PHC and MHC of HRQoL (66, 67).

In our sample, 49.75% of participants had more than one NCD, which is lower compared to what was reported by the nursing home residents in Armenia in earlier investigations (56). This difference can likely be attributed to age, as advancing age increases vulnerability to chronic conditions. A study conducted among adult refugees from Cuba, Iraq, and Afghanistan who arrived in the US found that 51.1% of refugees had only one NCD whereas 8.9% had two and 9.5% had three or more NCDs (68). Another study conducted among Syrian refugees in Jordan reported that 21.8% suffered from one NCD and 44.7% suffered from more than one NCD, which is quite similar to the rates obtained from our sample (69). Refugees who had more than one NCD reported lower PHC and MHC scores than those who had none in our study. This finding aligns with the results of other reports in the literature (70). In addition to the increased discomfort caused by more than one NCD, people may experience fatigue and helplessness due to the management of multiple NCDs, taking different medications, and having various medical appointments, which may negatively associated with both PHC and MHC scores (71, 72).

About 24.87% of the sample reported having heart disease, which was associated with both PHC and MHC scores. The domains most significantly associated with heart disease were physical functioning, role limitation due to physical health, general health and role limitation due to emotional health. Heart diseases can significantly lower patients’ physical functioning due to fatigue, shortness of breath, and chest pain (73). Moreover, chest pain increases the risk of anxiety and depression, which might affect mental well-being (74).

Bone and joint diseases which were prevalent in 34.01% of the respondents were significantly associated with both PHC and MHC scores, linked to all domains except role limitation due to physical health, role limitation due to emotional problems, and emotional well-being. Rao et al. (75) found that participants who have arthritis have significantly low scores in all domains of HRQoL compared with those without arthritis. Bone and joint diseases can cause pain, which limits the ability to perform daily activities which leads to discomfort and low score in PHC (76).

In our study, the prevalence of respiratory diseases/asthma was 4.06%, which was associated with both the PHC and MHC of HRQoL. Notably, it was linked with pain in the physical component, as well as energy/fatigue and social functioning in the mental component. In a cross-sectional study among chronic obstructive pulmonary disease (COPD) patients aged 45 and older, Kharbanda et al. (77) found that HRQoL was impaired and worsened as the severity increased. Airway obstruction and dependence on medications contribute to shortness of breath and emotional distress, which restrict mobility, lower PHC, and negatively associated with MHC of HRQoL (78). Respiratory diseases can lead to the constant fatigue, anxiety and depression, which can be explained by dyspnea, limiting their abilities to perform physical activities, worsening mental health and emotional well-being decreasing overall HRQoL (79, 80).

The prevalence of diabetes in our study was 15.23%, which is higher than the prevalence of insulin (0.22%) and non-insulin (1.26%) dependent diabetes found among refugees in Greece (81). Diabetes was significantly associated with both PHC and MHC components of HRQoL in our study. It was related to nearly all domains of HRQoL, with the exception of pain, emotional well-being, and social functioning. The results of our study are in line with the cross-sectional study conducted among diabetic refugees in Gaza, which reported substantially reduced HRQoL, particularly among women and older individuals (82).

This study has several limitations. A cross-sectional study design restricted our ability to interpret the direction of association of several factors with HRQoL. In addition, the lack of longitudinal follow-up limits our ability to assess changes in HRQoL over time or to examine long-term trajectories following displacement. A longitudinal study, which could shed light on the causal links and also track the changes in HRQoL and other measures over time would be most useful. Also, some health and behavioral characteristics were assessed based on self-reported data, which might have resulted in recall bias and inaccuracies in data. In particular, information on NCDs was based on self-report and not clinically verified, which may have introduced measurement bias due to misclassification or undiagnosed conditions. Since our sample was not assembled via random sampling, self-selection bias, which might have resulted in disproportionate representation of refugees with poorer health status, cannot be ruled out, thereby limiting the generalizability of the findings. Furthermore, we did not adjust for possible clustering by place of residence. Participants living in the same location may share similar characteristics or exposures, which could have resulted in underestimated standard errors. Since we could not perform a formula-based sample size calculation before the data collection, the power could be limited for some of our analyses.

## Conclusion

Our study found that age, sleep disorders, and NCDs including respiratory diseases/asthma, bone/joint diseases and diabetes were significantly associated with PHC and MHC scores of HRQoL. Refugees who had more than one NCD reported lower scores of PHC and MHC of HRQoL compared to those who had no NCD. Governmental support and interventions targeting older refugees, those with sleep disorders, and more than one NCD might be beneficial. Policy responses may include strengthening primary healthcare services, regular health assessments, screenings for NCDs and NCD management, as well as psychological counseling could prevent the incidence and exacerbation of health issues among forcibly displaced people. Repeated follow-up assessments should monitor variations in both PHC and MHC scores among refugees, as they may vary over time in response to the challenges associated with displacement. Longitudinal monitoring of HRQoL would allow policymakers and service providers to better understand changing needs and adapt interventions over time as refugee circumstances evolve.

## Data Availability

The raw data supporting the conclusions of this article will be made available by the authors, without undue reservation.
